# Challenges in the Diagnosis and Management of Culture-Negative Infective Endocarditis in a 64-Year-Old Hemodialysis Patient

**DOI:** 10.7759/cureus.88226

**Published:** 2025-07-18

**Authors:** Tomoo Tsujihata, Hiroyuki Nagano, Ryuichi M Sada, Eri Iwai-Kanai, Kazuhiro Hatta

**Affiliations:** 1 Department of General Internal Medicine, Tenri Hospital, Tenri, JPN; 2 Department of Transformative Protection to Infectious Disease, Graduate School of Medicine, Osaka University, Osaka, JPN

**Keywords:** blood cultures, cardiogenic shock, culture-negative infective endocarditis, hemodialysis, timing of surgery

## Abstract

We report a case of culture-negative infective endocarditis in a 64-year-old hemodialysis patient who presented with fever and dyspnea. Despite empirical antibiotic therapy and negative blood cultures, transesophageal echocardiography later revealed aortic valve vegetation and destruction. The patient developed cardiogenic shock and died before surgery. This case illustrates the diagnostic challenges of culture-negative infective endocarditis, especially in high-risk populations. Early blood culture collection and continuous reassessment of surgical timing are crucial to improve outcomes.

## Introduction

Infective endocarditis (IE) remains a life-threatening condition with high morbidity and mortality, particularly in patients undergoing maintenance hemodialysis. The prevalence of IE among dialysis patients is approximately 50 times higher than that among the general population, owing to factors such as frequent vascular access, impaired immunity, and a high burden of comorbidities [1]. Blood cultures play a pivotal role in the diagnosis of IE, as microbial evidence constitutes one of the major Duke criteria [2]. The Duke criteria are standard diagnostic guidelines for IE, recently updated in 2023 to reflect advances in testing and imaging [2]. However, prior administration of antibiotics can result in culture-negative IE, complicating diagnosis and delaying appropriate treatment decisions [3].

Management of IE requires a multidisciplinary approach, incorporating antimicrobial therapy and timely surgical intervention when indicated. The European Society of Cardiology (ESC) and American Heart Association guidelines provide surgical indications based on hemodynamic status, embolic risk, and infection control; however, discrepancies remain regarding the optimal timing of surgery, especially in semi-urgent cases [4,5]. Recent studies suggest that early surgical intervention is associated with improved survival outcomes, but clinical decisions must often be individualized based on dynamic patient status and interdisciplinary consensus [6].

We report a fatal case of blood culture-negative IE in a patient on maintenance hemodialysis, in whom diagnosis and treatment were delayed due to prior antibiotic administration and challenges in determining surgical timing. This case underscores the importance of obtaining blood cultures prior to initiating antibiotics in high-risk patients and highlights the need for continuous reassessment of surgical indications during hospitalization.

## Case presentation

A 64-year-old man on maintenance dialysis, independent in performing his activities of daily living, was admitted to our facility with a three-day history of bilateral shoulder pain, fever, and dyspnea. The patient was initially treated for suspected pneumonia with ceftriaxone at the referring hospital; however, the fever persisted, leading to a referral to our institution. His medical history included hypertension, hyperuricemia, and diabetes mellitus with nephropathy, which had led to hemodialysis over the past six years.

His vital signs were as follows: body temperature, 38.7ºC; blood pressure, 125/51 mmHg; pulse rate, 110 beats/min; respiratory rate, 26 breaths/min; and oxygen saturation, 95% on 2 L/min of oxygen. He was alert and oriented, with clear speech. Physical examination revealed faint crackles at both lung bases with no cardiac murmur or peripheral manifestations of endocarditis. An arteriovenous fistula was located on the left forearm, with no warmth or tenderness. Initial laboratory investigations revealed leukocytosis, anemia, and significantly impaired renal function. The C-reactive protein level was markedly elevated, while the rheumatoid factor was negative. Laboratory results on admission and on Day 4, along with corresponding reference ranges, are summarized in Table 1. Transthoracic echocardiography showed severe aortic regurgitation, but did not indicate whether it was attributable to valve degeneration or vegetation, and whether the patient had a preexisting valvular disease was unclear. Contrast-enhanced computed tomography revealed no abscesses or infectious foci.

**Table 1:** Admission and Day 4 laboratory results with reference ranges. eGFR, estimated glomerular filtration rate. White blood cell count and C-reactive protein levels decreased from admission to Day 4, indicating a reduction in inflammation. Anemia persisted, and serum creatinine remained elevated on both days, as expected in a patient receiving maintenance hemodialysis.

Parameter	Admission	Day 4	Units	Reference range
White cell count	9.7	8.8	x10^9/L	4.0-11.0
Neutrophils	6.8	6.1	x10^9/L	2.0-7.0
Neutrophil percentage	71.0	70.0	%	50-70%
Hemoglobin	10.7	9.6	g/dL	13.8-17.2
Hematocrit	32.8	29.1	%	40-50%
Platelets	276	264	x10^9/L	150-400
Sodium	134	123	mmol/L	135-145
Potassium	4.1	5.0	mmol/L	3.5-5.0
Chloride	97	84	mmol/L	98-106
Aspartate aminotransferase	32	11	U/L	6-42
Alanine aminotransferase	19	31	U/L	6-55
Urea	25	57	mg/dL	7-20
Creatinine	6.8	12.2	mg/dL	0.6-1.2
eGFR	7.2	3.8	mL/min/1.73 m²	>60
Albumin	2.9	2.4	g/dL	3.5-5.0
C-reactive protein	27	11	mg/dL	0-10
Lactic acid	1.8	1.1	mmol/L	0.5-2.2
Rheumatoid factor	0.0	-	IU/mL	<15

**Figure 1:**
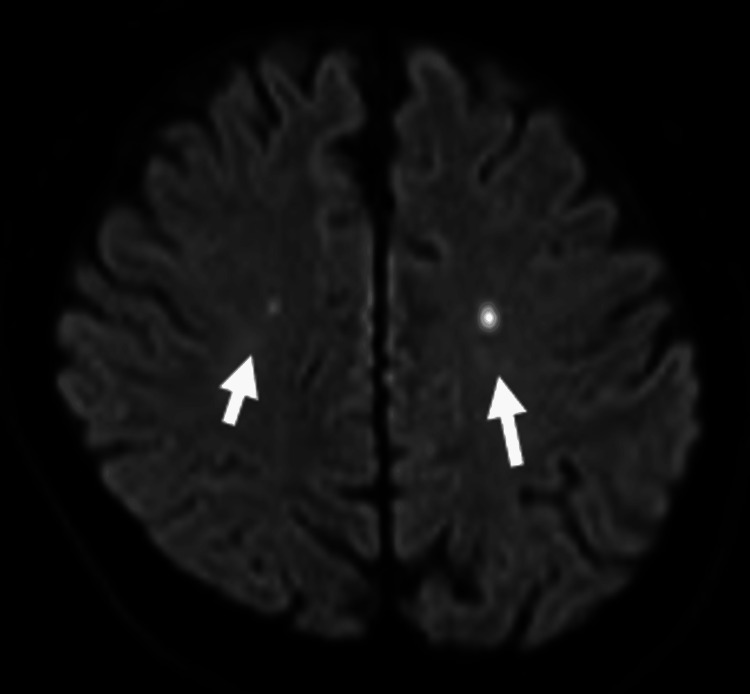
MRI of the head showing multiple acute cerebral microinfarctions (indicated by arrows). Diffusion-weighted magnetic resonance imaging of the patient’s head showing multiple acute cerebral microinfarctions caused by infective endocarditis.

Given the patient's clinical background and presentation, we consulted a cardiologist and suspected IE as the underlying cause of the fever. However, the blood cultures were negative, and we could not confirm the diagnosis. We started empirical administration of intravenous ceftriaxone and vancomycin, with plans for further evaluation and consideration of surgical intervention. Magnetic resonance imaging (MRI) on the fourth day of admission revealed multiple acute cerebral microinfarctions (Figure 1).

**Figure 2:**
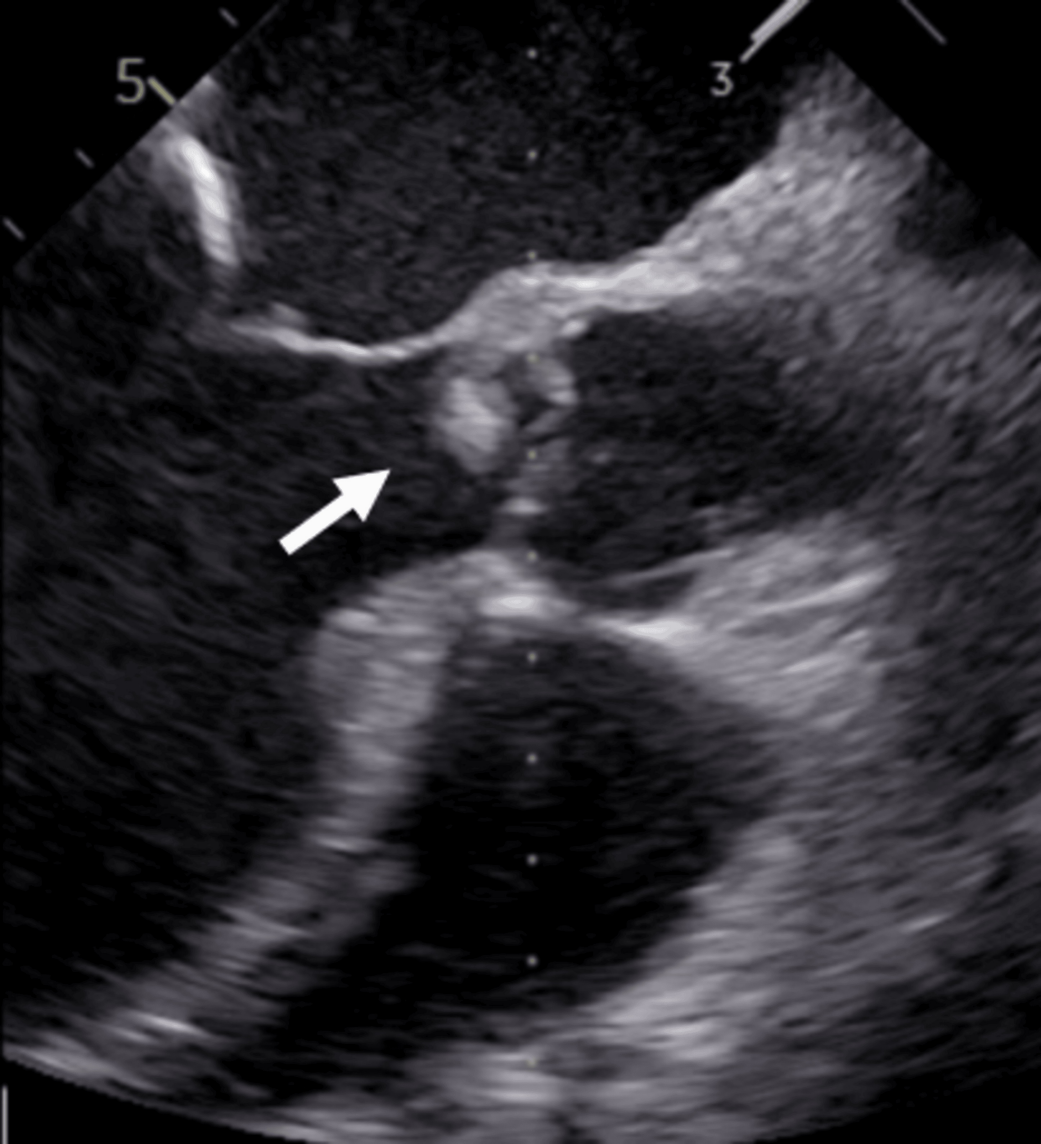
Aortic valve vegetation on TEE (indicated by arrow). TEE, transesophageal echocardiography. Transesophageal echocardiogram of a 64-year-old man showing vegetation on the aortic valve.

Four sets of blood cultures were negative on the first and third days of admission, likely owing to prior antibiotic administration at the referring hospital. The fever decreased, and inflammatory marker levels improved with antimicrobial therapy; however, persistent dyspnea necessitated ongoing oxygen support. During hemodialysis on the fifth day of admission, the patient’s blood pressure dropped to 70 mmHg within an hour of starting, and he was unable to continue dialysis due to unbearable general malaise. The same scenario was observed during hemodialysis the next day. Subsequent transesophageal echocardiography confirmed severe aortic regurgitation with attached vegetation (Figure 2).

A review of prior echocardiograms obtained at this point indicated no valvular abnormalities six months earlier, confirming new-onset aortic regurgitation around the time of this admission. Given the progressive clinical deterioration and hemodynamic instability, we consulted a cardiologist for emergent surgical intervention. Unfortunately, 30 minutes after this discussion, the patient experienced cardiopulmonary arrest. Transthoracic echocardiography revealed complete destruction of the aortic valve, leading to irreversible cardiogenic shock. Despite resuscitative efforts and initiation of venoarterial extracorporeal membrane oxygenation, the patient died on the eighth day of hospitalization.

## Discussion

We report a fatal case of a hemodialysis patient with blood culture-negative IE. The patient died despite antimicrobial therapy and a wait-and-see surgical approach. Through this case, two critical points are highlighted. First, the administration of antibiotics before drawing blood cultures can result in false-negative culture results, complicating the diagnosis of IE. This diagnostic uncertainty may delay appropriate treatment and hinder effective interdisciplinary discussions. This issue is particularly significant in patients with a high prevalence of bacteremia, such as those undergoing hemodialysis, underscoring the necessity of obtaining blood cultures before initiating antibiotic therapy. Second, although the current guidelines do not recommend emergent surgical intervention, there are instances in which a patient’s clinical condition necessitates a transition from non-emergent to emergent surgical intervention. Therefore, it is essential to continuously monitor the patient’s condition after hospitalization and regularly evaluate whether the timing of surgery is appropriate to ensure optimal treatment outcomes.

The administration of antibiotics before obtaining blood cultures, as in this case, can result in false-negative results, complicating the diagnosis of IE and hindering appropriate treatment decisions and interdisciplinary discussions. The most common cause of culture-negative IE is antibiotic administration before blood cultures are obtained [7]. In this case, the patient was suspected to have pneumonia and was treated with ceftriaxone by a previous physician, which is believed to have contributed to the culture-negative status. According to the Duke criteria, one of the major criteria for diagnosing IE is the detection of microorganisms consistent with IE in blood or tissue cultures [2]. Therefore, the present case did not meet this criterion. The fulfilled criteria included a major criterion of new severe aortic regurgitation detected on echocardiography and minor criteria such as fever above 38ºC and multiple arterial embolic strokes suspected from brain MRI. Therefore, this case was classified as "possible" rather than "definite" IE. Subsequent transesophageal echocardiography on Day 6 confirmed severe aortic regurgitation with attached vegetation, which led to a definitive diagnosis of IE. However, during the first few days of hospitalization, the case was classified only as "possible" IE. This initial diagnostic uncertainty impeded smooth discussions about the treatment strategy among the general internal medicine, cardiology, and cardiovascular surgery departments. Multidisciplinary management, including inputs from cardiology, surgery, and infectious disease specialists, is crucial for IE cases [4].

Failure to obtain blood cultures delays the diagnosis of IE, and culture-negative IE is associated with diagnostic uncertainty, making it difficult to use appropriate antibiotics and potentially worsening the prognosis compared with culture-positive cases [3]. Although it may be challenging to obtain blood cultures before antibiotic administration in all patients, blood cultures should be prioritized in patients at high risk of bacteremia, including those with suspected IE. Patients undergoing dialysis have a 50-fold higher prevalence of IE than the general population (estimated prevalence of 2.9%) [1]. In addition to dialysis, a history of intravenous drug abuse, prior heart valve surgery, and previously implanted cardiac devices are high-risk factors for IE [8]. According to the 2023 European Society of Cardiology guidelines, hemodialysis is a non-cardiac risk factor for IE [4]. For patients with a high pretest probability of IE, blood cultures should be aggressively obtained before antibiotic administration.

Although antibiotic treatment is initiated according to the guidelines with plans for elective surgery, the patient's condition may necessitate an urgent shift to surgery. Our patient presented with severe aortic regurgitation that had not progressed to uncontrollable pulmonary edema or shock. According to the ESC guidelines, this is classified as semi-urgent surgery [4]. However, discrepancies between guidelines regarding the timing of surgery for IE should be noted. While the European guidelines vaguely suggest "a few days" as the timeframe for surgery in semi-urgent cases, the American guidelines define early surgery as that which happens during the initial hospitalization, without providing specific guidance on when to switch to urgent surgery [5]. Recent studies have shown that patients undergoing early surgery have better survival outcomes than those receiving conservative antibiotic therapy or elective surgery [6]. Therefore, it is difficult to rely solely on a single guideline to determine the optimal timing for surgery. Given this complexity, the timing of surgery is often left to the discretion of cardiac surgeons in actual clinical practice. In this case, after discussions among the departments of general internal medicine, cardiology, and cardiovascular surgery, the decision was made to administer antibiotics before proceeding with surgery. Following antibiotic treatment, the patient's temperature normalized, inflammatory marker levels decreased, and the course appeared favorable. However, during dialysis on Day 5, the patient, who was scheduled to undergo three hours of ultrafiltration, experienced a drop in systolic blood pressure from the 90s to the 70s after just one hour, accompanied by generalized fatigue, leading to the cessation of ultrafiltration. Although the patient's blood pressure normalized after dialysis, the drop during the procedure suggested circulatory collapse [9]. The same issue occurred during dialysis the next day, and the patient experienced respiratory arrest the following day. At the time of the blood pressure drop during dialysis on Day 5, further dialysis should have been withheld, and the need for emergent surgery should have been re-discussed with the cardiology and cardiovascular surgery teams. Although the patient’s course seemed favorable, with the resolution of fever, a decrease in inflammatory markers, and persistently negative blood cultures after antibiotic therapy, the sudden deterioration in the patient’s condition and eventual death highlight the difficulty in determining the optimal timing for surgery when clinical progress seems favorable under medical management. Continuous assessment of the patient’s overall condition after admission is crucial, with constant consideration of the appropriate timing of surgery.

## Conclusions

This case highlights the clinical complexity of managing culture-negative infective endocarditis in high-risk patients, such as those on long-term hemodialysis. Delayed diagnosis due to prior antibiotic administration and challenges in determining surgical timing can significantly affect outcomes. Our experience emphasizes the critical importance of obtaining blood cultures prior to initiating antimicrobial therapy and maintaining a dynamic approach to treatment planning. Even when clinical parameters appear stable under medical therapy, close monitoring and timely reassessment of surgical indications are essential to prevent unexpected deterioration. A multidisciplinary, timely approach is key to improving outcomes in similar cases.
